# Avoiding fusion plasma tearing instability with deep reinforcement learning

**DOI:** 10.1038/s41586-024-07024-9

**Published:** 2024-02-21

**Authors:** Jaemin Seo, SangKyeun Kim, Azarakhsh Jalalvand, Rory Conlin, Andrew Rothstein, Joseph Abbate, Keith Erickson, Josiah Wai, Ricardo Shousha, Egemen Kolemen

**Affiliations:** 1 Department of Mechanical and Aerospace Engineering, Princeton University, Princeton, NJ USA; 2 Department of Physics, Chung-Ang University, Seoul, South Korea; 3 Princeton Plasma Physics Laboratory, Princeton, NJ USA; 4 Department of Astrophysical Sciences, Princeton University, Princeton, NJ USA

**Keywords:** Nuclear fusion and fission, Magnetically confined plasmas, Mechanical engineering, Computational science, Information theory and computation

## Abstract

For stable and efficient fusion energy production using a tokamak reactor, it is essential to maintain a high-pressure hydrogenic plasma without plasma disruption. Therefore, it is necessary to actively control the tokamak based on the observed plasma state, to manoeuvre high-pressure plasma while avoiding tearing instability, the leading cause of disruptions. This presents an obstacle-avoidance problem for which artificial intelligence based on reinforcement learning has recently shown remarkable performance^1–4^. However, the obstacle here, the tearing instability, is difficult to forecast and is highly prone to terminating plasma operations, especially in the ITER baseline scenario. Previously, we developed a multimodal dynamic model that estimates the likelihood of future tearing instability based on signals from multiple diagnostics and actuators^5^. Here we harness this dynamic model as a training environment for reinforcement-learning artificial intelligence, facilitating automated instability prevention. We demonstrate artificial intelligence control to lower the possibility of disruptive tearing instabilities in DIII-D^6^, the largest magnetic fusion facility in the United States. The controller maintained the tearing likelihood under a given threshold, even under relatively unfavourable conditions of low safety factor and low torque. In particular, it allowed the plasma to actively track the stable path within the time-varying operational space while maintaining H-mode performance, which was challenging with traditional preprogrammed control. This controller paves the path to developing stable high-performance operational scenarios for future use in ITER.

## Abstract (web summary)

Artificial intelligence control is used to avoid the emergence of disruptive tearing instabilities in the magnetically confined fusion plasma in the DIII-D tokamak reactor.

## Main

As the demand for energy and the need for carbon neutrality continue to grow, nuclear fusion is rapidly emerging as a promising energy source in the near future due to its potential for zero-carbon power generation, without creating high-level waste. Recently, the nuclear fusion experiment accompanied by 192 lasers at the National Ignition Facility successfully produced more energy than the injected energy, demonstrating the feasibility of net energy production^7^. Tokamaks, the most studied concept for the first fusion reactor, have also achieved remarkable milestones: The Korea Superconducting Tokamak Advanced Research sustained plasma at ion temperatures hotter than 100 million kelvin for 30 seconds^8^, a plasma remained in a steady state for 1,000 seconds in the Experimental Advanced Superconducting Tokamak^9^, and the Joint European Torus broke the world record by producing 59 megajoules of fusion energy for 5 seconds^10,11^. ITER, the world’s largest science project with the collaboration of 35 nations, is under construction for the demonstration of a tokamak reactor^12^.

Although fusion experiments in tokamaks have achieved remarkable success, there still remain several obstacles that we must resolve. Plasma disruption is one of the most critical issues to be solved for the successful long-pulse operation of ITER^13^. Even a few plasma disruption events can induce irreversible damage to the plasma-facing components in ITER. Recently, techniques for predicting disruption using artificial intelligence (AI) have been demonstrated in multiple tokamaks^14,15^, and mitigation of the damage during disruption is being studied^16,17^. Tearing instability, the most dominant cause of plasma disruption^18^, especially in the ITER baseline scenario^19^, is a phenomenon where the magnetic flux surface breaks due to finite plasma resistivity at rational surfaces of safety factor *q* = *m*/*n*. Here, *m* and *n* are the poloidal and toroidal mode numbers, respectively. In modern tokamaks, the plasma pressure is often limited by the onset of neoclassical tearing instability because the perturbation of pressure-driven (so-called bootstrap) current becomes a seed for it^20^. Research on the evolution and suppression of existing tearing instabilities using actuators has been widely conducted^21–27^. However, the tearing instability induces unrecoverable energy loss and often leads to disruption before being suppressed in the ITER baseline condition, where the edge safety factor (*q*_95_) and plasma rotation are low^19^. Therefore, we need to ‘avoid’ the onset of tearing instability, not suppress it after it appears. To avoid its occurrence, physics research is also underway to investigate the onset cause or seed of instability^28–30^. However, calculating tearing stability requires massive computational simulations based on resistive magnetohydrodynamics or gyrokinetics, which are not suitable for real-time stability prediction and control during experiments. This suggests the need for AI-accelerated real-time instability-avoidance techniques.

**Fig. 1:**
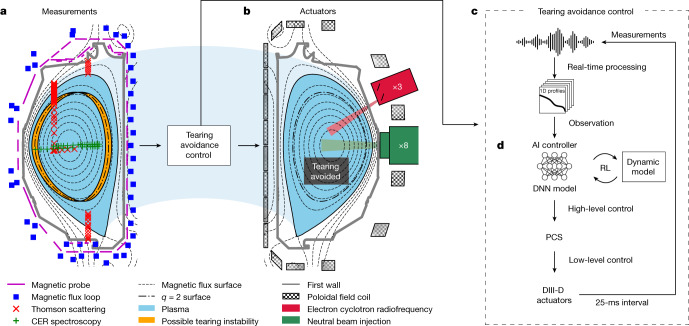
The overall architecture of the tearing-avoidance system in the DIII-D tokamak. **a**, The selected diagnostic systems used in this work: magnetics, Thomson scattering (TS) and charge-exchange recombination (CER) spectroscopy. The possible tearing instability of *m*/*n* = 2/1 is shown in orange. **b**, The heating, current drive and control actuators used in this work. **c**, Schematic description of the tearing-avoidance control, including preprocessing, high-level control by a DNN and low-level control by a PCS. **d**, The AI controller based on the DNN.

The deep reinforcement learning (RL) technique has shown remarkable performance in nonlinear, high-dimensional actuation problems^1^. Moreover, it has shown notable advantages in avoidance control problems^2^, which is essentially similar to the objective of this work. Recently, RL has been applied to tokamak control and optimization, showing promising achievements^3,4,31–35^. The RL algorithm optimizes the actor model based on a deep neural network (DNN), and the actor model gradually learns the action policy leading to higher rewards in a given environment. By specifically designing the reward function, we can train the actor model to actively control the tokamak to pursue a high-pressure plasma while keeping the tearing possibility low. An essential component of RL is the training environment, which can interact with the actor model by responding to its action. For the training environment, we employ a dynamic model that predicts future plasma pressure and tearing likelihood (so-called tearability) developed in ref. ^5^. In this work, we develop an AI controller that adaptively controls actuators to pursue high plasma pressure while maintaining low tearability, based on observed plasma profiles. The overall architecture of this tearing-avoidance system is depicted in Fig. 1.

Figure 1a,b shows an example plasma in DIII-D and selected diagnostics and actuators for this work. A possible tearing instability of *m*/*n* = 2/1 at the flux surface of *q* = 2 is also illustrated. Figure 1c shows the tearing-avoidance control system, which maps the measurement signals and the desired actuator commands. The signals from different diagnostics have different dimensions and spatial resolutions, and the availability and target positions of each channel vary depending on the discharge condition. Therefore, the measured signals are preprocessed into structured data of the same dimension and spatial resolution using the profile reconstruction^36–38^ and equilibrium fitting (EFIT)^39^ before being fed into the DNN model. The DNN-based AI controller (Fig. 1d) determines the high-level control commands of the total beam power and plasma shape based on the trained control policy. Its training using RL is described in the following section. The plasma control system (PCS) algorithm calculates the low-level control signals of the magnetic coils and the powers of individual beams to satisfy the high-level AI controls, as well as user-prescribed constraints. In our experiments, we constrain *q*_95_ and total beam torque in the PCS to maintain the ITER baseline-similar condition where tearing instability is crucial.

## RL design for tearing-avoidance control

**Fig. 2:**
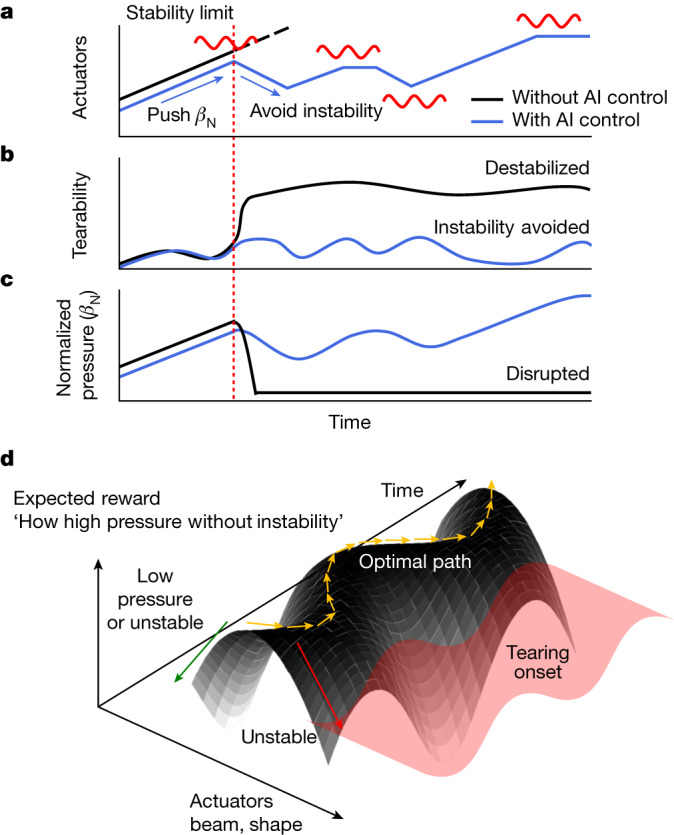
Illustration of the tokamak control by the AI tearing-avoidance system and the plasma responses. **a**, The time evolution of actuators with (blue) and without (black) the AI control. Possible tearing stability limits are indicated in red. **b**, The tearability expected by actuators' control. **c**, The normalized plasma pressure expected by actuators' control. **d**, The expected plasma evolution by the desired AI control in parametric space.

For efficient fusion power generation, it is essential to maintain high plasma pressure without disruptive instability. However, as external heating like neutral beams increases the plasma pressure, a stability limit is eventually reached, as shown by the black lines in Fig. 2a, beyond which the tearing instability is excited. The instability can induce plasma disruption shortly, as shown in Fig. 2b,c. Moreover, this stability limit varies depending on the plasma state, and lowering the pressure can also cause instability under certain conditions^19^. As depicted by the blue lines in Fig. 2, the actuators can be actively controlled depending on the plasma state to pursue high plasma pressure without crossing the onset of instability.

This is a typical obstacle-avoidance problem, where the obstacle here has a high potential to terminate the operation immediately. We need to control the tokamak to guide the plasma along a narrow acceptable path where the pressure is high enough and the stability limit is not exceeded. To train the actor model for this goal with RL, we designed the reward function, *R*, to evaluate how high pressure the plasma is under tolerable tearability, as shown in equation (1). *β*_N_ represents the normalized plasma pressure, *T* is the tearability and *k* is the prescribed threshold. Here, *β*_N_ and *T* are the predictions after 25 ms resulting from the action of the AI controller. The prediction of future *β*_N_ and *T* using a dynamic model is described in more detail in Methods. The threshold *k* is set to 0.2, 0.5 or 0.7 in this work. If the predicted tearability is below a given threshold, the actor receives a positive reward based on the attained plasma pressure, and it receives a negative reward otherwise.

1 $$R({\beta }_{{\rm{N}}},T;k)=\left\{\begin{array}{ll}{\beta }_{{\rm{N}}},\quad  & {\rm{if}}\,T < k\\ k-T,\quad  & {\rm{otherwise}}\end{array}\right.$$

To obtain a higher reward, defined in equation (1), the actor should first increase *β*_N_ through its control actions. However, higher *β*_N_ tends to make the plasma unstable, causing the tearability (*T*) to exceed the threshold (*k*) at some point, which in turn reduces the reward. We note that the reward shows a steep change when *T* exceeds *k*, like a binary penalty. This leads the actor model to prioritize maintaining *T* below *k* over increasing *β*_N_. After sufficient training with RL, the actor can determine the control actions that pursue high plasma pressure while keeping the tearability below the given threshold. This control policy enables the tokamak operation to follow a narrow desired path during a discharge, as illustrated in Fig. 2d. It is noted that the reward contour surface in Fig. 2d is a simplified representation for illustrative purposes, while the actual reward contour according to equation (1) has a sharp bifurcation near the tearing onset.

The action variables controlled by AI are set as the total beam power and the plasma triangularity. Although there are other controllable actuators through the PCS, such as the beam torque, plasma current or plasma elongation, they strongly affect *q*_95_ and the plasma rotation. Thus, for the purpose of maintaining the ITER baseline-similar condition of *q*_95_ ≈ 3 and beam torque ≤1 Nm, these other actuators were fixed during the experiments.

**Fig. 3:**
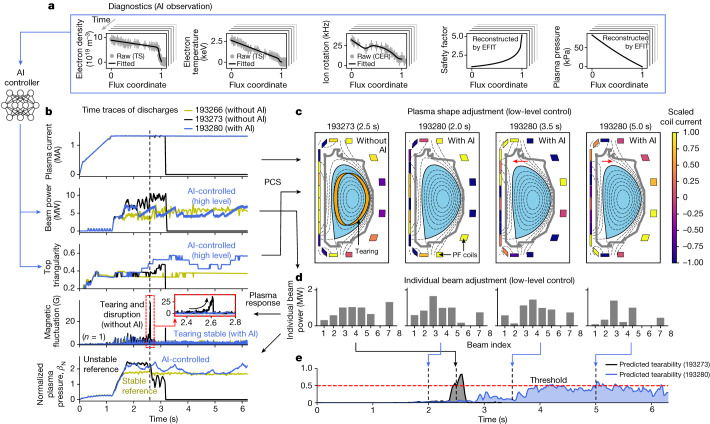
The AI-based tearing-avoidance experiments in DIII-D. **a**, The observation of the AI controller; the preprocessed profiles of electron density, electron temperature, ion rotation, safety factor and plasma pressure. **b**, The time traces of discharges 193266 (stable reference), 193273 (unstable reference) and 193280. Discharge 193280 is the AI-controlled one. **c**, The low-level coil current control by the PCS and the plasma boundary variation. Scaled currents of poloidal field (PF) coils are shown in colour. **d**, The low-level individual beam power control by the PCS. **e**, The estimated tearability for discharges 193273 and 193280.

The observation variables are set as one-dimensional kinetic and magnetic profiles mapped in a magnetic flux coordinate because the tearing onset strongly depends on their spatial information and gradients^19^. Specifically, the actor observes profiles of the electron density, electron temperature, ion rotation, safety factor and plasma pressure. An example set of observation profiles is shown in Fig. 3a.

## Tearing-avoidance control in DIII-D

An example of plasma disruption due to tearing instability is depicted by the black lines (discharge 193273) in Fig. 3b. In discharge 193273, a traditional feedback control (not AI control) was applied to maintain *β*_N_ = 2.3. However, at *t* = 2.6 s, a large tearing instability occurred, as shown in the fourth row of Fig. 3b. This led to unrecoverable degradation of *β*_N_, eventually resulting in a disruption at *t* = 3.1 s. This indicates that the tearing onset boundary is crossed at some point before *t* = 2.6 s. Figure 3e depicts the post-experiment tearability prediction for this discharge. This post-analysis reveals that the tearing event could have been forecasted as early as 200 ms beforehand, providing sufficient time to lower tearability via appropriate control. As the model predicts the onset of tearing instability, not classifies whether the current state is tearing or not, the tearability decreases back to 0 after the onset passes (*t* > 2.7 s). The yellow line (discharge 193266) in Fig. 3b, which targets *β*_N_ = 1.7 under traditional control, represents a stable example that could roughly be considered as a conservative bound for tearing stability.

In discharge 193280 (the blue lines in Fig. 3b), beam power and plasma triangularity were adaptively controlled via AI. Here the AI controller was trained to ensure that the predicted tearability does not exceed 0.5 (*k* = 0.5 in equation (1)). As shown in the second and third rows of Fig. 3b, the AI controller actively adjusts the two actuators according to the time-evolving plasma state. Other controllable parameters were kept fixed during discharge to constrain *q*_95_ ≈ 3 and beam torque ≤1 Nm. At each time point, the AI controller observes the plasma profiles and determines control commands for beam power and triangularity. The PCS algorithm receives these high-level commands and derives low-level actuations, such as magnetic coil currents and the individual powers of the eight beams^39–41^. The coil currents and resulting plasma shape at each phase are shown in Fig. 3c and the individual beam power controls are shown in Fig. 3d.

The blue line in Fig. 3e, a post-experiment estimation for the AI-controlled discharge (193280), shows that the estimated tearability is maintained just below the given threshold until the end, reflecting the exact intention in equation (1). This experiment demonstrated the ability to achieve lower tearability than the traditional control discharge 193273, and higher time-integrated performance than 193266, through adaptive and active control via AI.

The control policy of a trained actor model can vary depending on the threshold (*k*) of the reward function equation (1) during the RL training. As the tearability threshold for receiving negative rewards increases, the control policy becomes less conservative. The controller trained with a higher threshold is willing to tolerate higher tearability while pushing *β*_N_.

**Fig. 4:**
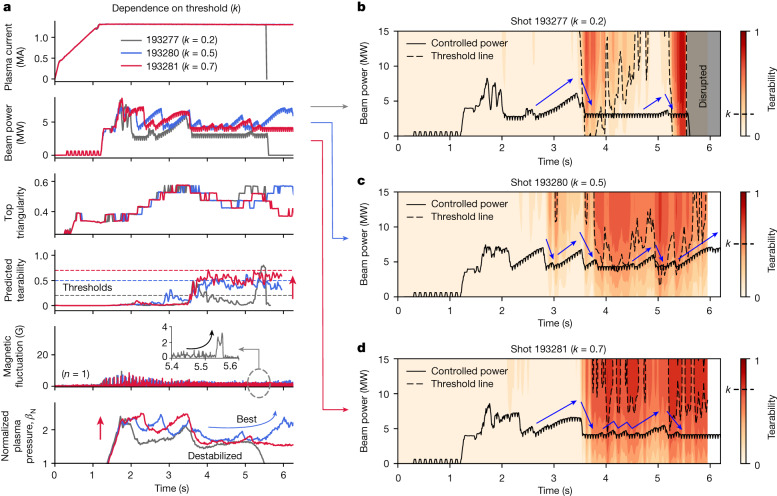
Comparison of the experiments conducted with different threshold settings. **a**, The time traces of discharges with different thresholds; 193277 (*k* = 0.2), 193280 (*k* = 0.5) and 193281 (*k* = 0.7). **b**–**d**, The actual beam power and the contour of the predicted tearability for possible beam powers in the three discharges 193277 (**b**), 193280 (**c**) and 193281 (**d**).

Figure 4a shows three experiments conducted by controllers of different threshold values. Discharges 193277 (grey), 193280 (blue) and 193281 (red) correspond to threshold values of 0.2, 0.5 and 0.7, respectively. In the cases of *k* = 0.5 and *k* = 0.7, the plasma is sustained without disruptive instability until the preprogrammed end of the flat top. Figure 4b–d shows the post-calculated tearability for the three discharges. The background contour colour in each graph represents the predicted tearability for possible beam powers at each time point, and the actual beam power is indicated by the black line. The dashed lines correspond to the tearability contour lines for each threshold (0.2, 0.5 or 0.7).

Different threshold values result in different characteristics during the AI control in the experiments. In the early phase (*t* < 3.5 s), the high-threshold controller (*k* = 0.7) tends to push *β*_N_ harder, as shown in the last row of Fig. 4a. However, this leads to putting the plasma in a more unstable region and accepting higher tearability around 0.7 after *t* = 3.5 s, and the increased tearability does not decrease afterwards. In contrast, the low-threshold controller (*k* = 0.2) is overly conservative and suppresses the possibility of instability too much in the early phase. The AI control maintained a very low tearability of less than 0.2 until *t* = 5 s, but a large instability, difficult to be avoided, suddenly occurred at *t* = 5.5 s. As revealed in the post-analysis (Fig. 4b), the tearing prediction model could forecast the instability 300 ms before the disruption, and the controller also attempted to further reduce the beam power accordingly. However, as the beam power had already reached its prescribed lower bound, it could not be lowered further, ultimately failing to avoid the instability. The lower bound of the beam power was prescribed to prevent L-mode back transition, independent of the RL control, and this was not considered during the training of the controller. As *k* = 0.2 is a conservative setting, the controller often attempts to reduce the beam power, which frequently hits the lower bound. As a result, the control interference due to the preset lower bound led to the failure of tearing avoidance. In contrast, the controller with a moderate threshold (*k* = 0.5) sustains the plasma until the end of the flat top and eventually recovers *β*_N_ again. Therefore, an optimal threshold value is required to maintain stable plasma for a long time. In Fig. 4c, the AI controller of *k* = 0.5 actively tries to avoid touching the threshold through proactive control before the instability warning. Because the reward in equation (1) is computed using the tearability 25 ms after the controller’s action at each time point, the trained controller takes actions tens of milliseconds before a warning occurs.

## Discussion

We present a technique for avoiding disruptive tearing instability in a tokamak using the RL method. The AI-based tearing-avoidance system actively controls the beam power and the plasma triangularity to maintain the possibility of future tearing-instability occurrence at a low level. This enabled maintaining the tearability below the threshold under the low-*q*_95_ and low-torque conditions in DIII-D. In addition, our controller has demonstrated the ability to robustly avoid tearing instability not only in a specific experimental condition like the ITER baseline condition but also in other operational environments and even in accidental cases, which is further discussed in Methods.

Our work is a proof-of-concept study on tearing avoidance using RL and is still in the early stages of fine-tuning. For more useful applications, further experiments and fine-tuning are required. Nonetheless, this work demonstrates the capability that RL could be applied to real-time control of core plasma physics, as well as plasma boundary control shown in ref. ^3^. We also note that this demonstration is a successful extension of machine-learning capability in the fusion area, bringing insight and a path to developing the integrated control for high-performance operational scenarios in future tokamak devices, beyond the single instability control. There are further potential applications of the tearing-avoidance control developed in this work. For example, this algorithm can be combined with the plasma profile prediction system^42^ or physics information^43^, which enables optimizing the entire discharge through combined autoregressive prediction of the plasma state and desired actuator control. In addition, by sustaining plasmas without disruption under extreme conditions, we can discover phenomena such as a new kind of self-generated current^44^, which may help us to achieve efficient fusion energy harvesting.

## Methods

### DIII-D

The DIII-D National Fusion Facility, located at General Atomics in San Diego, USA, is a leading research facility dedicated to advancing the field of fusion energy through experimental and theoretical research. The facility is home to the DIII-D tokamak, which is the largest and most advanced magnetic fusion device in the United States. The major and minor radii of DIII-D are 1.67 m and 0.67 m, respectively. The toroidal magnetic field can reach up to 2.2 T, the plasma current is up to 2.0 MA and the external heating power is up to 23 MW. DIII-D is equipped with high-resolution real-time plasma diagnostic systems, including a Thomson scattering system^45^, charge-exchange recombination^46^ spectroscopy and magnetohydrodynamics reconstruction by EFIT^37,39^. These diagnostic tools allow for the real-time profiling of electron density, electron temperature, ion temperature, ion rotation, pressure, current density and safety factor. In addition, DIII-D can perform flexible total beam power and torque control through reliable high-frequency modulation of eight different neutral beams in different directions. Therefore, DIII-D is an optimal experimental device for verifying and utilizing our AI controller that observes the plasma state and manipulates the actuators in real time.

### Plasma control system

One of the unique features of the DIII-D tokamak is its advanced PCS^47^, which allows researchers to precisely control and manipulate the plasma in real time. This enables researchers to study the behaviour of the plasma under a wide range of conditions and to test ideas for controlling and stabilizing the plasma. The PCS consists of a hierarchical structure of real-time controllers, from the magnetic control system (low-level control) to the profile control system (high-level control). Our tearing-avoidance algorithm is also implemented in this hierarchical structure of the DIII-D PCS and is integrated with the existing lower-level controllers, such as the plasma boundary control algorithm^39,41^ and the individual beam control algorithm^40^.

### Tearing instability

Magnetic reconnection refers to the phenomenon in magnetized plasmas where the magnetic-field line is torn and reconnected owing to the diffusion of magnetic flux (*ψ*) by plasma resistivity. This magnetic reconnection is a ubiquitous event occurring in diverse environments such as the solar atmosphere, the Earth’s magnetosphere, plasma thrusters and laboratory plasmas like tokamaks. In nested magnetic-field structures in tokamaks, magnetic reconnection at surfaces where *q* becomes a rational number leads to the formation of separated field lines creating magnetic islands. When these islands grow and become unstable, it is termed tearing instability. The growth rate of the tearing instability classically depends on the tearing stability index, *Δ*′, shown in equation (2).

2 $${\varDelta }^{{\prime} }\equiv {\left[\frac{1}{\psi }\frac{{\rm{d}}\psi }{{\rm{d}}x}\right]}_{x=0-}^{x=0+}$$

where *x* is the radial deviation from the rational surface. When *Δ*′ is positive, the magnetic topology becomes unstable, allowing (classical) tearing instability to develop. However, even when *Δ*′ is negative (classical tearing instability does not grow), ‘neoclassical’ tearing instability can arise due to the effects of geometry or the drift of charged particles, which can amplify seed perturbations. Subsequently, the altered magnetic topology can either saturate, unable to grow further^48,49^, or can couple with other magnetohydrodynamic events or plasma turbulence^50–53^. Understanding and controlling these tearing instabilities is paramount for achieving stable and sustainable fusion reactions in a tokamak^54^.

### ITER baseline scenario

The ITER baseline scenario (IBS) is an operational condition designed for ITER to achieve fusion power of *P*_fusion_ = 500 MW and a fusion gain of *Q* ≡ *P*_fusion_/*P*_external_ = 10 for a duration of longer than 300 s (ref. ^12^). Compared with present tokamak experiments, the IBS condition is notable for its considerably low edge safety factor (*q*_95_ ≈ 3) and toroidal torque. With the PCS, DIII-D has a reliable capability to access this IBS condition compared with other devices; however, it has been observed that many of the IBS experiments are terminated by disruptive tearing instabilities^19^. This is because the tearing instability at the *q* = 2 surface appears too close to the wall when *q*_95_ is low, and it easily locks to the wall, leading to disruption when the plasma rotation frequency is low. Therefore, in this study, we conducted experiments to test the AI tearability controller under the conditions of *q*_95_ ≈ 3 and low toroidal torque (≤1 Nm), where the disruptive tearing instability is easy to be excited.

However, in addition to the IBS where the tearing instability is a critical issue, there are other scenarios, such as hybrid and non-inductive scenarios for ITER^12^. These different scenarios are less likely to disrupt by tearing, but each has its own challenges, such as no-wall stability limit or minimizing inductive current. Therefore, it is worth developing further AI controllers trained through modified observation, actuation and reward settings to address these different challenges. In addition, the flexibility of the actuators and sensors used in this work at DIII-D will differ from that in ITER and reactors. Control policies under more limited sensing and actuation conditions also need to be developed in the future.

### Dynamic model for tearing-instability prediction

**Extended Data Fig. 1:**
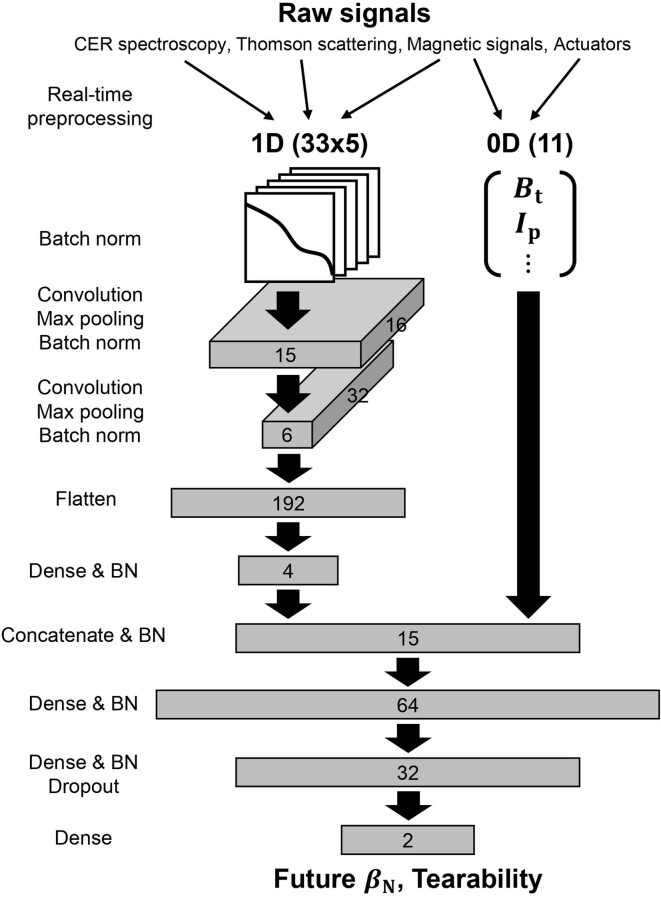
The DNN architecture of the dynamic model that predicts future tearability. The inputs of the dynamic model are the 1-dimensional signals of the plasma state and the scalar signals of the proposed actuators. The outputs are the normalized plasma pressure (*β*_N_) and the tearability metric after 25 ms.

To predict tearing events in DIII-D, we first labelled whether each phase was tearing-stable or not (0 or 1) based on the *n* = 1 Mirnov coil signal in the experiment. Using this labelled experimental data, we trained a DNN-based multimodal dynamic model that receives various plasma profiles and tokamak actuations as input and predicts the 25-ms-after tearing likelihood as output. The trained dynamic model outputs a continuous value between 0 and 1 (so-called tearability), where a value closer to 1 indicates a higher likelihood of a tearing instability occurring after 25 ms. The architecture of this model is shown in Extended Data Fig. 1. The detailed descriptions for input and output variables and hyperparameters of the dynamic prediction model can be found in ref. ^5^. Although this dynamic model is a black box and cannot explicitly provide the underlying cause of the induced tearing instability, it can be utilized as a surrogate for the response of stability, bypassing expensive real-world experiments. As an example, this dynamic model is used as a training environment for the RL of the tearing-avoidance controller in this work. During the RL training process, the dynamic model predicts future *β*_N_ and tearability from the given plasma conditions and actuator values determined by the AI controller. Then the reward is estimated based on the predicted state using equation (1) and provided to the controller as feedback.

Figure 4b–d shows the contour plots of the estimated tearability for possible beam powers at the given plasma conditions of our control experiments. The actual beam power controlled by the AI is indicated by the black solid lines. The dashed lines are the contour line of the threshold value set for each discharge, which can roughly represent the stability limit of the beam power at each point. The plot shows that the trained AI controller proactively avoids touching the tearability threshold before the warning of instability.

**Extended Data Fig. 2:**
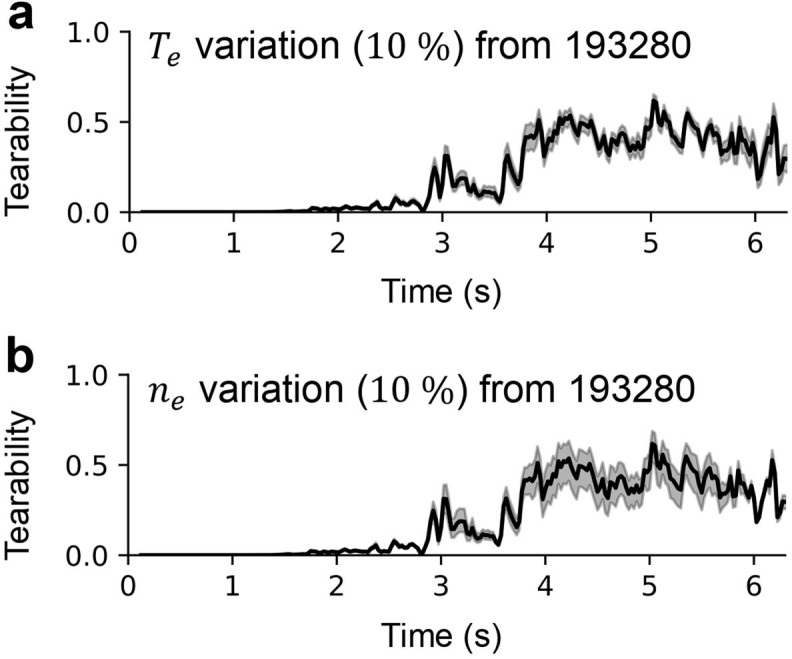
The sensitivity of the tearability against the diagnostic errors in 193280. **a**, The evolution of tearability with uncertainty range caused by the electron temperature error of 10 %. **b**, The evolution of tearability with uncertainty range caused by the electron density error of 10 %.

The sensitivity of the tearability against the diagnostic errors of the electron temperature and density is shown in Extended Data Fig. 2. The filled areas in Extended Data Fig. 2 represent the range of tearability predictions when increasing and decreasing the electron temperature and density by 10%, respectively, from the measurements in 193280. The uncertainty in tearability due to electron temperature error is estimated to be, on average, 10%, and the uncertainty due to electron density error is about 20%. However, even when considering diagnostic errors, the trend in tearing stability over time can still be observed to remain consistent.

### RL training for tearing avoidance

The dynamic model used for predicting future tearing-instability dynamics is integrated with the OpenAI Gym library^55^, which allows it to interact with the controller as a training environment. The tearing-avoidance controller, another DNN model, is trained using the deep deterministic policy gradient^56^ method, which is implemented using Keras-RL (https://keras.io/)^57^.

**Extended Data Fig. 3:**
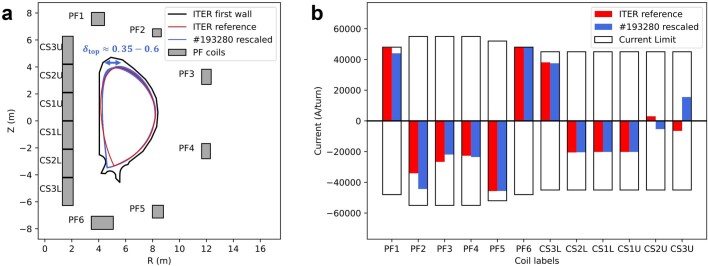
The ITER-rescaled plasma boundary of discharge 193280 and the required poloidal field coil currents. **a**, The poloidal cross-section of the ITER first wall, plasma boundaries, and PF coils. The blue shade is the range of the ITER-rescaled plasma boundary of discharge 193280 and the red line is the ITER reference plasma boundary. **b**, The maximum coil current required to shape each plasma boundary compared to the coil current limits. The PF coils of ITER can support the new plasma boundary shape determined by AI.

The observation variables consist of 5 different plasma profiles mapped on 33 equally distributed grids of the magnetic flux coordinate: electron density, electron temperature, ion rotation, safety factor and plasma pressure. The safety factor (*q*) can diverge to infinity at the plasma boundary when the plasma is diverted. Therefore, 1/*q* has been used for the observation variables to reduce numerical difficulties^42^. The action variables include the total beam power and the triangularity of the plasma boundary, and their controllable ranges were limited to be consistent with the IBS experiment of DIII-D. The AI-controlled plasma boundary shape has been confirmed to be achievable by the poloidal field coil system of ITER, as shown in Extended Data Fig. 3.

**Extended Data Fig. 4:**
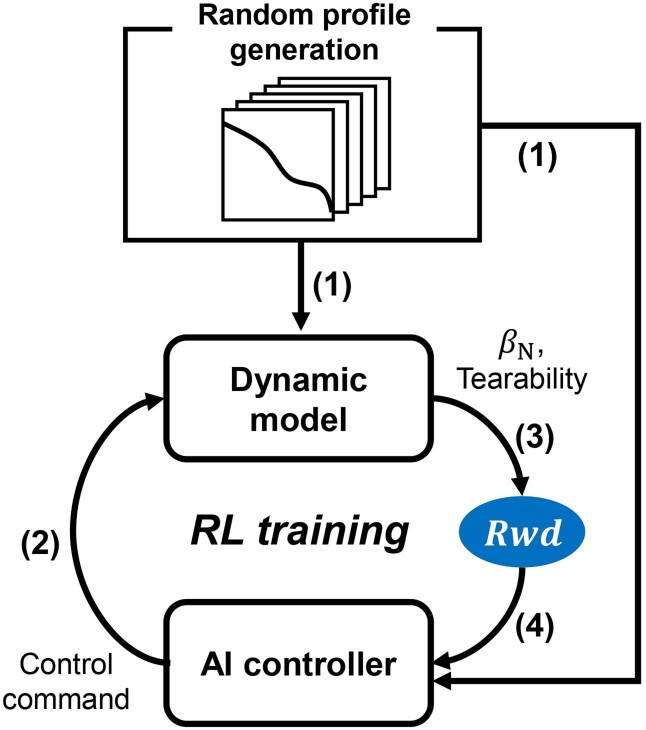
The pipeline of the RL training used in our work. First, random plasma profiles are selected from experimental data to be fed to both the dynamic model and the AI controller. The AI controller observes the plasma profiles and determines the action. Then, the dynamic model predicts the future *β*_N_ and tearability. Lastly, the reward is estimated from the predicted state to optimize the AI controller.

The RL training process of the AI controller is depicted in Extended Data Fig. 4. At each iteration, the observation variables (five different profiles) are randomly selected from experimental data. From this observation, the AI controller determines the desirable beam power and plasma triangularity. To reduce the possibility of local optimization, action noises based on the Ornstein–Uhlenbeck process are added to the control action during training. Then the dynamic model predicts *β*_N_ and tearability after 25 ms based on the given plasma profiles and actuator values. The reward is evaluated according to equation (1) using the predicted states, and then given as feedback for the RL of the AI controller. As the controller and the dynamic model observe plasma profiles, it can reflect the change of tearing stability even when plasma profiles vary due to unpredictable factors such as wall conditions or impurities. In addition, although this paper focuses on IBS conditions where tearing instability is critical, the RL training itself was not restricted to any specific experimental conditions, ensuring its applicability across all conditions. After training, the Keras-based controller model is converted to C using the Keras2C library^58^ for the PCS integration.

Previously, a related work^17^ employed a simple bang-bang control scheme using only beam power to handle tearability. Although our control performance may seem similar to that work in terms of *β*_N_, it is not true if considering other operating conditions. In ITER and future fusion devices, higher normalized fusion gain (*G* ∝ *Q*) with stable core instability is critical. This requires a high *β*_N_ and small *q*_95_ as $G\propto {\beta }_{{\rm{N}}}/{q}_{95}^{2}$. At the same time, owing to limited heating capability, high *G* has to be achieved with weak plasma rotation (or beam torque). Here, high *β*_N_, small ${q}_{95}^{2}$ and low torque are all destabilizing conditions of tearing instability, highlighting tearing instability as a substantial bottleneck of ITER.

**Extended Data Fig. 5:**
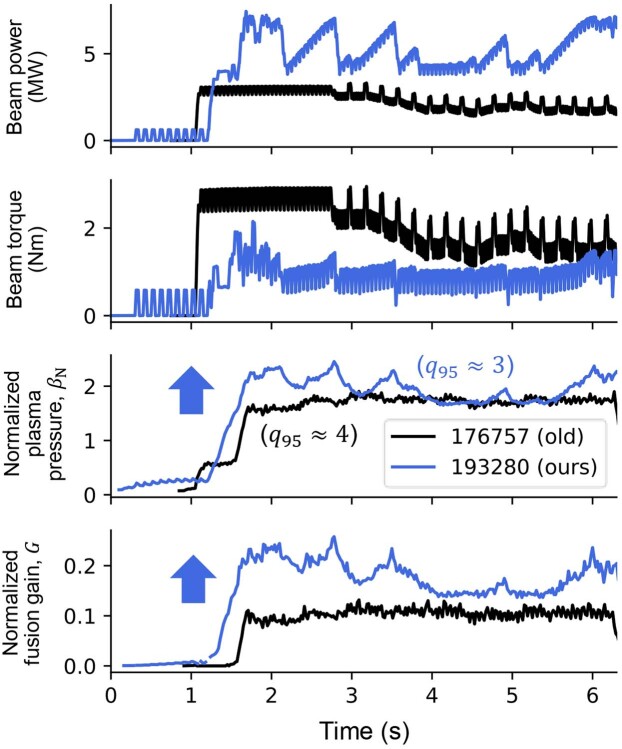
Comparison of the discharge using a previous controller (176757) and our controlled one (193280). Multi-actuator multi-objectives control could achieve higher *β*_*N*_ and *G* under more unfavorable condition. Here, the time domain for 176757 was shifted by + 0.75 s to synchronize the H-mode onset between two shots.

As shown in Extended Data Fig. 5, our control achieves a tearing-stable operation of much higher *G* than the test experiment shown in ref. ^17^. This is possible by maintaining higher (or similar) *β*_N_ with lower *q*_95_ (4 → 3), where tearing instability is more likely to occur. In addition, this is achieved with a much weaker torque, further highlighting the capability of our RL controller in harsher conditions. Therefore, this work shows more ITER-relevant performance, providing a closer and clearer path to the high fusion gain with robust tearing avoidance in future devices.

**Extended Data Fig. 6:**
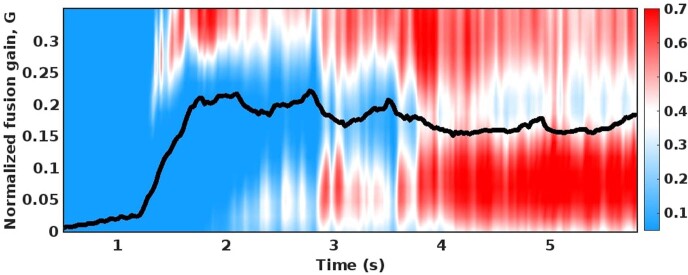
Time trace of the normalized fusion gain for discharge 193280, where contour color illustrates the tearability. The RL control successfully drives plasma through the valley of tearability.

In addition, the performance of RL control in achieving high fusion can be further highlighted when considering the non-monotonic effect of *β*_N_ on tearing instability. Unlike *q*_95_ or torque, both increasing and decreasing *β*_N_ can destabilize tearing instabilities. This leads to the existence of optimal fusion gain (as *G* ∝ *β*_N_), which enables the tearing-stable operation and makes system control more complicated. Here, Extended Data Fig. 6 shows the trace of RL-controller discharge in the space of fusion gain versus time, where the contour colour illustrates the tearability. This clearly shows that the RL controller successfully drives plasma through the valley of tearability, ensuring stable operation and showing its remarkable performance in such a complicated system.

Such a superior performance is feasible by the advantages of RL over conventional approaches, which are described below.

1. By employing a ‘multi-actuator (beam and shape) multi-objectives (low tearability and high *β*_N_)’ controller using RL, we were able to enter a higher*-β*_N_ region while maintaining tolerable tearability. As shown in Extended Data Fig. 5, our controlled discharge (193280) shows a higher *β*_N_ and *G* than the one in the previous work (176757). This advantage of our controller is because it adjusts the beam and plasma shape simultaneously to achieve both increasing *β*_N_ and lowering tearability. It is notable that our discharge has more unfavourable conditions (lower *q*_95_ and lower torque) in terms of both *β*_N_ and tearing stability.
2. The previous tearability model evaluates the tearing likelihood based on current zero-dimensional measurements, not considering the upcoming actuation control. However, our model considers the one-dimensional detailed profiles and also the upcoming actuations, then predicts the future tearability response to the future control. This can provide a more flexible applicability in terms of control. Our RL controller has been trained to understand this tearability response and can consider future effects, while the previous controller only sees the current stability. By considering the future responses, ours offers a more optimal actuation in the longer term instead of a greedy manner.

**Extended Data Fig. 7:**
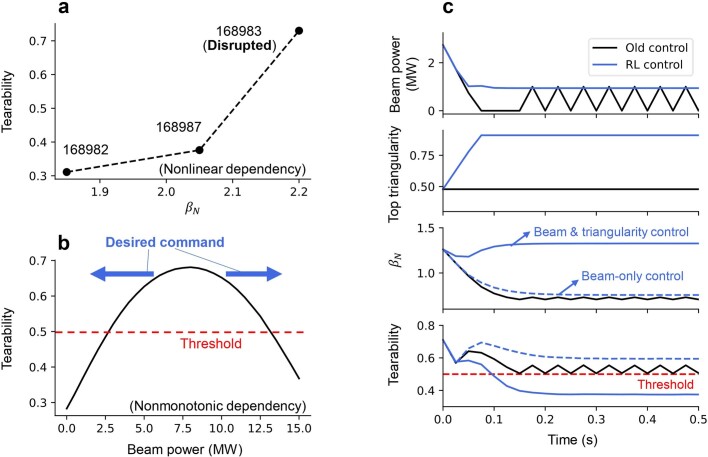
Non-monotonic dependence of tearability and its effect on control. **a**, Non-linear dependence of tearability on *β*_*N*_ observed in experiments. **b**, Non-monotonic dependence of tearability on beam power observed in model predictions. **c**, Comparison of a simple bang-bang controller (black) and our controller (blue) in a simulative plasma. While the simple controller induces an oscillatory actuation, our controller could achieve swifter stabilization with higher *β*_*N*_. The plasma response without adjusting triangularity from the RL control is also shown with blue dashed lines.

This enables the application in more generic situations beyond our experiments. For instance, as shown in Extended Data Fig. 7a, tearability is a nonlinear function of *β*_N_. In some cases (Extended Data Fig. 7b), this relation is also non-monotonic, making increasing the beam power the desired command to reduce tearability (as shown in Extended Data Fig. 7b with a right-directed arrow). This is due to the diversity of the tearing-instability sources such as *β*_N_ limit, *Δ*′ and the current well. In such cases, using a simple control shown in ref. ^17^ could result in oscillatory actuation or even further destabilization. In the case of RL control, there is less oscillation and it controls more swiftly below the threshold, achieving a higher *β*_N_ through multi-actuator control, as shown in Extended Data Fig. 7c.

### Control of plasma triangularity

Plasma shape parameters are key control knobs that influence various types of plasma instability. In DIII-D, the shape parameters such as triangularity and elongation can be manipulated through proximity control^41^. In this study, we used the top triangularity as one of the action variables for the AI controller. The bottom triangularity remained fixed across our experiments because it is directly linked to the strike point on the inner wall.

We also note that the changes in top triangularity through AI control are quite large compared with typical adjustments. Therefore, it is necessary to verify whether such large plasma shape changes are permitted for the capability of magnetic coils in ITER. Additional analysis, as shown in Extended Data Fig. 3, confirms that the rescaled plasma shape for ITER can be achieved within the coil current limits.

### Robustness of maintaining tearability against different conditions

**Extended Data Fig. 8:**
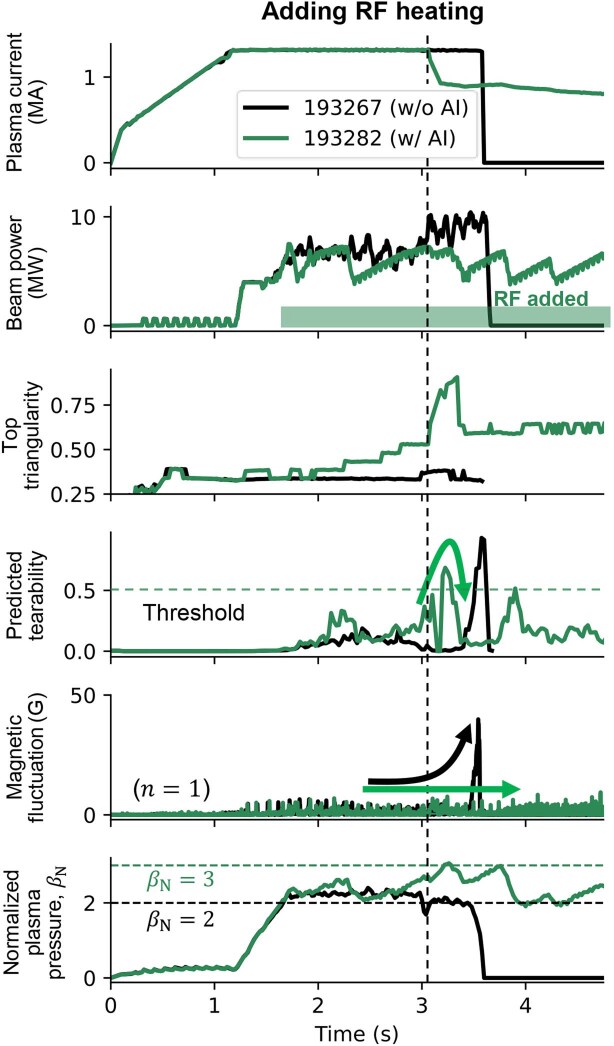
Control experiments under the different plasma conditions by adding RF heating. In the AI-controlled discharge (193282), the plasma current control is suddenly lost at *t* = 3.1 s, but the tearability control is still working after that.

The experiments in Figs. 3b and 4a have shown that the tearability can be maintained through appropriate AI-based control. However, it is necessary to verify whether it can robustly maintain low tearability when additional actuators are added and plasma conditions change. In particular, ITER plans to use not only 50 MW beams but also 10–20 MW radiofrequency actuators. Electron cyclotron radiofrequency heating directly changes the electron temperature profile and the stability can vary sensitively. Therefore, we conducted an experiment to see whether the AI controller successfully maintains low tearability under new conditions where radiofrequency heating is added. In discharge 193282 (green lines in Extended Data Fig. 8), 1.8 MW of radiofrequency heating is preprogrammed to be steadily applied in the background while beam power and plasma triangularity are controlled via AI. Here, the radiofrequency heating is towards the core of the plasma and the current drive at the tearing location is negligible.

However, owing to the sudden loss of plasma current control at *t* = 3.1 s, *q*_95_ increased from 3 to 4, and the subsequent discharge did not proceed under the ITER baseline condition. It should be noted that this change in plasma current control was unintentional and not directly related to AI control. Such plasma current fluctuation sharply raised the tearability to exceed the threshold temporarily at *t* = 3.2 s, but it was immediately stabilized by continued AI control. Although it is eventually disrupted owing to insufficient plasma current by the loss of plasma current before the preprogrammed end of the flat top, this accidental experiment demonstrates the robustness of AI-based tearability control against additional heating actuators, a wider *q*_95_ range and accidental current fluctuation.

In normal plasma experiments, control parameters are kept stationary with a feed-forward set-up, so that each discharge is a single data point. However, in our experiments, both plasma and control are varying throughout the discharge. Thus, one discharge consists of multiple control cycles. Therefore, our results are more important than one would expect compared with standard fixed control plasma experiments, supporting the reliability of the control scheme.

**Extended Data Fig. 9:**
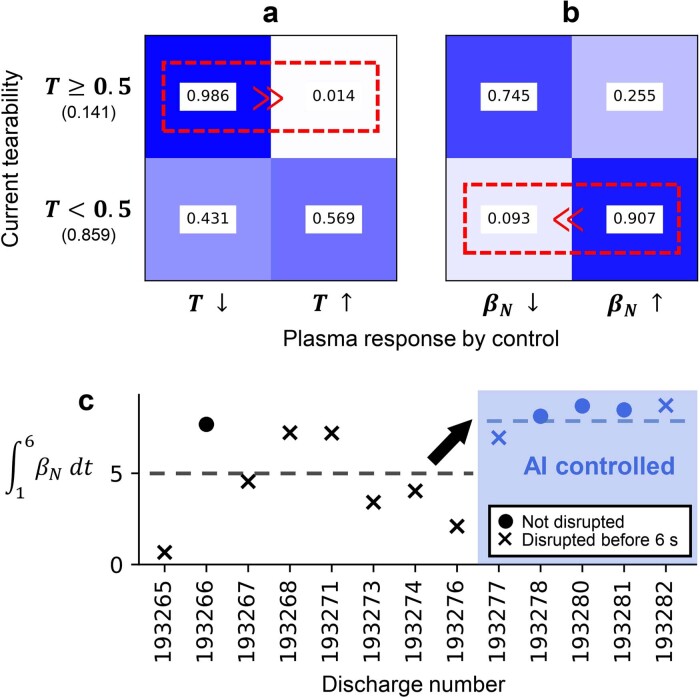
Statistics of the predicted plasma response by RL control in the existing database. **a**, The response of tearability by control when the original plasma was unstable (top) and stable (bottom). **b**, The response of *β*_*N*_ by control when the original plasma was unstable (top) and stable (bottom). **c**, Change of the time-integrated *β*_*N*_ after the RL control during our experimental session, where circles represent non-disrupted shots, while crosses indicate disrupted ones. After the RL controller was applied, the average time-integrated *β*_*N*_ increased, and the disrupted rate decreased.

In addition, the predicted plasma response due to RL control for 1,000 samples randomly selected from the experimental database, which includes not just the IBS but all experimental conditions, is shown in Extended Data Fig. 9a,b. When *T* > 0.5 (unstable, top), the controller tries to decrease *T* rather than affecting *β*_N_, and when *T* < 0.5 (stable, bottom), it tries to increase *β*_N_. This matches the expected response by the reward shown in equation (1). In 98.6% of the unstable phase, the controller reduced the tearability, and in 90.7% of the stable phase, the controller increased *β*_N_.

**Extended Data Fig. 10:**
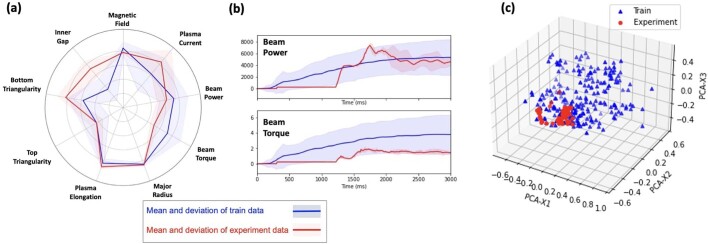
Comparison of several input data of our experiments with the training database distribution. **a**, Radar chart of the major input features distribution space, for the training data (blue) and our experiments (red). **b**, Time trace of the distribution of selected actuators. **c**, PCA analysis of the multi-dimensional input data distribution.

Extended Data Fig. 9c shows the achieved time-integrated *β*_N_ for the discharge sequences of our experiment session. Discharges until 193276 either did not have the RL control applied or had tearing instability occurring before the control started, and discharges after 193277 had the RL control applied. Before RL control, all shots except one (193266: low-*β*_N_ reference shown in Fig. 3b) were disrupted, but after RL control was applied, only two (193277 and 193282) were disrupted, which were discussed earlier. The average time-integrated *β*_N_ also increased after the RL control. In addition, the input feature ranges of the controlled discharges are compared with the training database distribution in Extended Data Fig. 10, which indicates that our experiments are neither too centred (the model not overfitted to our experimental condition) nor too far out (confirming the availability of our controller on the experiments).

## Online content

Any methods, additional references, Nature Portfolio reporting summaries, source data, extended data, supplementary information, acknowledgements, peer review information; details of author contributions and competing interests; and statements of data and code availability are available at 10.1038/s41586-024-07024-9.

## Data Availability

The data that support the findings of this study are available from the corresponding author upon reasonable request.
